# Risk factors for bloodstream infection (BSI) in patients with severe acute respiratory distress syndrome (ARDS) supported by veno–venous extracorporeal membrane oxygenation (VV–ECMO)

**DOI:** 10.1186/s12890-022-02164-y

**Published:** 2022-09-28

**Authors:** Liuting Yang, Min Li, Sichao Gu, Yingying Feng, Xu Huang, Yi Zhang, Ye Tian, Xiaojing Wu, Qingyuan Zhan, Linna Huang

**Affiliations:** 1 NHC Key Laboratory of Pneumoconiosis, Shanxi Province Key Laboratory of Respiratory, Department of Pulmonary and Critical Care Medicine, The First Hospital of Shanxi Medical University, Taiyuan, Shanxi People’s Republic of China; 2 Department of Pulmonary and Critical Care Medicine, National Center of Respiratory Medicine, China–Japan Friendship Hospital, No.2 Yinghua East Road, Chaoyang District, Beijing, 100029 People’s Republic of China

**Keywords:** Acute respiratory distress syndrome, Extracorporeal membrane oxygenation, Bloodstream infection, Risk factors

## Abstract

**Background:**

There were relatively few studies about the incidence and risk factors for bloodstream infection (BSI) in patients with severe acute respiratory distress syndrome (ARDS) supported by veno–venous extracorporeal membrane oxygenation (VV–ECMO).

**Methods:**

Patients who were diagnosed with severe ARDS and received VV–ECMO treatment in the medical intensive care unit of China–Japan Friendship Hospital from August 2013 to March 2019 were retrospectively studied. The pathogens isolated from blood culture (BC) were identified and analyzed for drug sensitivity. The risk factors for BSI were analyzed by logistic regression.

**Results:**

A total of 105 patients were included in this single–center retrospective cohort study. Among them, 23 patients (22%) had BSIs. 19 cases were identified as primary BSI; while the other 4 cases were as secondary BSI. A total of 23 pathogenic strains were isolated from BCs, including gram–negative (G^–^) bacilli in 21 (91%) cases, gram–positive (G^+^) cocci in 1 case, fungus in 1 case, and multidrug–resistant (MDR) organisms in 8 cases. Compared with patients without BSI, patients with BSI had a higher Murray score (odds ratio = 6.29, *P* = 0.01) and more blood transfusion (odds ratio = 1.27, *P* = 0.03) during ECMO.

**Conclusions:**

The incidence of BSI in patients with severe ARDS supported by VV–ECMO was 22%. G^–^ bacilli was the main pathogen, and most of them were MDR–G^–^ bacilli (MDR–GNB). Higher Murray score and more blood transfusion may be the independent risk factors for BSI.

## Introduction

Veno–venous extracorporeal membrane oxygenation (VV–ECMO) is the gold standard of support in the treatment of severe refractory acute respiratory distress syndrome (ARDS). Although equipment technology has greatly improved, the incidence of complications, especially infection, remains high and might affect the clinical outcomes of patients.

According to two retrospective analyses of Extracorporeal Life Support Organization (ELSO) data by Vogel and Bizzarro, the prevalence of hospital–acquired infections in adult patients during ECMO was 21% [1], while the incidence of BSI during ECMO ranged from 3 to 18% [2–5]. Compared with that in patients supported by veno–arterial ECMO (VA–ECMO), the infection rate in patients supported by VV–ECMO was higher [6]. The reasons might be attributed to longer treatment times and more frequent exposure to antibiotics and systemic steroids during VV–ECMO.

The pathogens and risk factors for BSI vary among different patients and different modes of ECMO. *Enterobacteriaceae* was reported to be the most common pathogen of BSIs, accounting for 16%, and the incidence of BSI caused by *Enterobacteriaceae* was 4.45 cases/1000 ECMO days [2]. Renal failure [7] and blood transfusion due to anemia and thrombocytopenia were identified as risk factors for BSI in patients with cancer [8]. A study in 92 patients with VA–ECMO indicated that age and serum total bilirubin level pre–ECMO were risk factors [9]. A single–center retrospective cohort study noted that patients with BSI during VV–ECMO had a longer duration of ECMO support than patients without BSI (18 days vs. 9 days, *P* < 0.01) [10]. The effect of BSI on mortality in ECMO patients remains controversial. A retrospective cohort study by Steiner et al. observed a threefold increase in the risk of death in patients suffering from BSI during ECMO [11]. However, other studies revealed that although BSI increased the length of hospitalization, there was no effect on hospital mortality [1–4].

Relevant data of patients with severe pneumonia–induced ARDS who are supported by VV–ECMO are extremely limited. We therefore conducted a study to evaluate the incidence and risk factors for BSI in severe ARDS patients supported by VV–ECMO.

## Methods

### Study design and participants

From August 2013 to March 2019, 276 patients treated with ECMO were admitted to the MICU of China–Japan Friendship Hospital. Among them, 243 patients were supported with VV–ECMO, 30 patients with VA–ECMO and 3 patients with ECCO_2_R.

### Inclusion criteria

1. Severe ARDS (PaO_2_/FiO_2_ ≤ 100 mmHg when positive end-expiratory pressure (PEEP) ≥ 5 mmHg) due to pneumonia;
2. ECMO initiation within 7 days after mechanical ventilation;
3. VV–ECMO support for more than 24 h.

### Exclusion criteria

1. Age < 18 years old;
2. VA–ECMO and ECCO_2_R.

### Methods and parameters of ECMO

All patients were punctured with the Seldinger technique. The centrifugal pump and CPB pipeline were from the ROTAFLOW and PLS CPB pipeline systems of MAQUET Company in Sweden. Puncture catheters were acquired from MAQUET or Medtronic (USA). The femoral vein was the drainage end and the jugular vein was the reflux end. Peripheral ECMO cannulas were almost always sutured at the insertion site along the ECMO line to fix the cannulas with tube–holder devices or sutured directly to the skin and covered with sterile dressing.

### Plan for prevention and treatment of nosocomial infections

Since 2013, we have implemented a standardized nosocomial infection prevention plan, which includes using an alcohol disinfectant to wash hands, changing dressings at the puncture point of the ECMO cannula every three days, and preventing ventilator–associated pneumonia (wearing isolation clothing before any operation; keeping patients in a 30–45° semi reclined position; administering chlorhexidine at least twice a day for oral care; and performing airtight endotracheal intubation). No antibiotic prophylaxis or anti–infective treatment was administered to ECMO patients with definite infection.

### Blood culture collection protocol

Blood collectors disinfected their hands before collection and wore disposable gloves or sterile gloves of appropriate size. After decontamination of the skin and lids of bottles with 75% ethanol, natural drying was performed for 60 s. Blood (10 mL) from the peripheral vein was collected in one aerobic and anaerobic bottle and shaken vigorously to mix well.

### Data collection

The clinical trial observation form (CRF) of ECMO patients was completed and entered into the electronic database. The variables included age, sex, body mass index (BMI), past medical history, immunosuppressive status, causes of ARDS, main indications for ECMO, severity of disease before ECMO [including Acute Physiology and Chronic Health Evaluation II (APACHE II), Sequential Organ Failure Assessment (SOFA), Predicting Death for Severe ARDS on VV⁃ECMO (PRESERVE), Respiratory ECMO Survival Prediction (RESP), and Acute Lung Injury scores]. In addition, related laboratory tests were performed during ECMO. Ventilator parameters, mechanical ventilation time before ECMO, ECMO duration, acute kidney injury (AKI) during ECMO, continuous renal replacement therapy (CRRT) during ECMO, massive hemorrhage during ECMO, type of blood product and volume of blood transfused during ECMO, length of intensive care unit (ICU) stay, total length of hospital stay and outcomes of patients were recorded.

### Definition of BSI

BSI was defined as two separate blood cultures (BCs) positive for the same pathogenic organism in addition to signs of infection, including leukocytosis, leukopenia, fever or hypothermia, according to the definitions of the Centers for Disease Control/National Healthcare Safety Network [12–14]. *Corynebacterium* spp., *Bacillus* spp., *Cutibacterium* spp., coagulase–negative Staphylococci (CoNS), viridians group *Streptococci*, *Aerococcus* spp. and *Micrococcus* spp. were considered common skin contaminants unless the same bacterial strain was isolated from two separate BCs within 48 h of each other [14].

BCs were performed when BSI was suspected between 24 h after ECMO and within 48 h after ECMO withdrawal, and the causative organism of BSI was recorded. When the organism isolated from the BCs was unrelated to infection at another site or when the BSI was related to catheter use, the infection was classified as primary BSI [12]; when the organism isolated from BC was identical to an organism identified from another site, the infection was classified as secondary BSI. Only the first BSI was recorded.

A sudden increase in the leukocyte count, purulent secretion in the airway, the drainage of pus from an open wound, fever, new pulmonary consolidation or diffuse exudative shadows in both lungs on dynamic chest radiography, low perfusion, insufficient oxygen delivery, etc., are all manifestations of BSI. It is recommended that patients receiving ECMO support for more than 2 weeks should be regularly cultured to monitor for BSI.

### Definition of a massive hemorrhage event

Significant bleeding was defined as a hemoglobin decrease of more than 20 g/L within 24 h, blood loss of more than 20 ml/kg/d or red blood cell (RBC) demand more than 10 ml/kg within 24 h. Retroperitoneal hemorrhage, pulmonary hemorrhage, central nervous system hemorrhage, or hemorrhage requiring surgical intervention were also considered massive hemorrhage events.

### Definition of immunosuppressed status

Immunosuppressed status was defined as organ transplantation, immunosuppressant usage, or use of prednisolone at a dosage of over 15 mg for longer than 1 month [15].

### Statistical methods

Data were analyzed using SPSS (version 25.0) software (SPSS Inc., Chicago, IL, USA). Normally distributed continuous variables are expressed as the $\overline{x }$±standard deviation (SD) and were compared using the t test. Nonnormally distributed continuous variables are expressed as medians and quartiles and were compared using the Wilcoxon rank–sum test. Categorical variables were compared using the *x*^2^ test. *P* values < 0.05 were considered significant.

Variables with significant differences and that were considered to be of clinical significance were entered into the logistic regression analysis. Logistic regression analysis with 95% confidence intervals (95% CIs) and odds ratios (ORs) was used to evaluate independent risk factors for BSI.

## Results

**Fig. 1:**
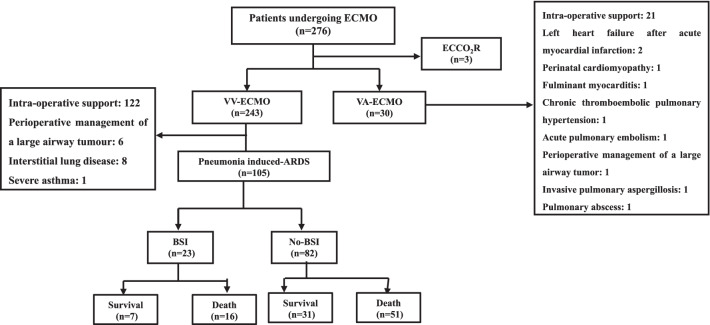
Flow diagram of this study. From August 2013 to March 2019, 276 patients receiving ECMO were admitted to the MICU. Among them, 243 patients were supported with VV–ECMO, 30 patients with VA–ECMO and 3 patients with ECCO_2_R. A total of 105 patients who met the inclusion criteria were enrolled. According to the BSI criteria, 23 patients were diagnosed with BSI, while the other 82 patients were diagnosed with non–BSI. Of the 23 patients with BSI, 7 survived, and 16 died

A total of 276 patients were screened for enrollment, and 171 patients who did not meet the eligibility criteria were excluded. The flow of this study is presented in a diagram (Fig. 1).

### Incidence of and pathogens causing BSI

BSI occurred in 23 of 105 patients (22%). The time interval between the initiation of ECMO support and BSI was 6.5 days (median; interquartile range [IQR]: 4.0, 18.5). There were 19(83%) cases of primary BSI and 4 (17%) cases of secondary BSI. Among the primary BSIs, 4 cases were catheter–related infections (CRIs). The cause of secondary BSI was pneumonia. In total, 21 strains of G^–^–rods were isolated, including *Acinetobacter baumannii* in 11 cases [carbapenem–resistant *Acinetobacter baumannii* (CRAB) in 6 cases and multidrug–resistant *Acinetobacter baumannii* (MDRAB) in 5 cases], *Burkholderia cepacian* in 5 cases, *Pseudomonas aeruginosa* in 2 cases [multidrug–resistant *Pseudomonas aeruginosa* (MDRPA) in 1 case], and *Klebsiella pneumoniae* in 3 cases [carbapenem–resistant *Enterobacteriaceae* (CRE) in 2 cases and multidrug–resistant *Enterobacteriaceae* (MDRE) in 1 case]. One strain of G^+^ cocci (*Enterococcus faecium*) and 1 strain of fungi (*Candida parapsilosis*) were identified. The rate of resistance of *Acinetobacter baumannii* to cephalosporins and carbapenems was 54.5%, and the rate of resistance to sulfamethoxazole trimethoprim was 81.8%. Only 7 of these infection patients survived, and the mortality rate (70%) was high.

### Comparison between the BSI group and the non–BSI group (***Table ******1***)

**Table 1:** Comparison of patients with and without BSI during VV–ECMO

Variables	All (*n* = 105)	BSI (*n* = 23)	Non–BSI (*n* = 82)	*P* value
*Basic information and personal history*				
Age (years)	49 ± 16	48 ± 19	51 ± 16	0.56
Male (*n*, %)	76 (72%)	21 (91%)	55 (67%)	0.02*
BMI (kg/m^2^)	24.8 ± 4.7	25.1 ± 4.7	24.7 ± 4.7	0.57
History of smoking (*n*, %)	43 (41%)	8 (35%)	35 (43%)	0.34
*Past medical history*				
History of diabetes (*n*, %)	20 (19%)	3 (13%)	17 (21%)	0.41
History of hypertension (*n*, %)	35 (33%)	12 (52%)	23 (28%)	0.03*
History of chronic lung diseases (*n*, %)	12 (11%)	1 (4%)	11 (13%)	0.23
Malignant tumor (*n*, %)	3 (3%)	0 (0%)	3 (4%)	0.35
Immunosuppressed status (*n*, %)	28 (27%)	7 (30%)	21 (26%)	0.64
*Causes of pneumonia*				
Bacterial (*n*, %)	30 (29%)	7 (30%)	23 (28%)	0.82
Virus (*n*, %)	54 (51%)	15 (66%)	39 (47%)	0.13
Fungal (*n*, %)	18(17%)	1(4%)	17 (21%)	0.07
Special pathogen infection (*n*, %)	3 (3%)	0 (0%)	3 (4%)	0.35
*Severity pre–ECMO*				
APACHE II score	18 ± 6	19 ± 6	18 ± 7	0.42
SOFA score	9 ± 4	9 ± 4	8 ± 4	0.23
PRESERVE score	5 ± 3	4 ± 3	5 ± 2	0.36
RESP score	2 (–1–4)	2 (0–3)	2 (–1–4)	0.75
Murray score	3.3 ± 0.5	3.5 ± 0.5	3.3 ± 0.5	0.02*
*Laboratory examinations*				
PH	7.3 ± 0.1	7.3 ± 0.1	7.3 ± 0.1	0.21
PaO_2_/FiO_2_ (mmHg)	73.1 ± 29.2	80.7 ± 31.8	77.1 ± 31.8	0.67
PCO_2_ (mmHg)	54.5 ± 25.2	58.3 ± 23.7	53.6 ± 26.1	0.44
Lac (mmol/L)	1.9 (1.5–2.6)	2.2 (1.5–3.7)	1.9 (1.4–2.4)	0.28
MAP (mmHg)	79 ± 17	78 ± 18	80 ± 17	0.52
WBC (× 10^9^/L)	11.8 (7.7–20.4)	10.9 (5.9–18.2)	12.3 (8.3–19.0)	0.29
NEU (%)	89.6 ± 7.5	90.8 ± 5.4	89.5 ± 7.8	0.49
LY (× 10^9^/L)	0.5 (0.3–1.0)	0.5 (0.3–0.8)	0.6 (0.3–1.1)	0.52
PCT (ng/ml)	1.2 (0.5–6.4)	3.4 (0.9–6.5)	0.8 (0.4–5.3)	0.09
*Organ support during ECMO*				
IPPV (*n*, %)	100 (95%)	23 (100%)	77 (94%)	0.23
Vasopressors (*n*, %)	89 (85%)	20 (87%)	69 (84%)	0.74
CRRT (*n*, %)	51 (49%)	16 (70%)	35 (43%)	0.02*
Massive hemorrhage events (*n*, %)	42 (40%)	8 (35%)	34 (42%)	0.56
*Blood transfusion during ECMO*				
Transfusion (*n*, %)	57 (54%)	17 (74%)	40 (49%)	0.03*
*Cumulative volume*				
RBCs (u)	2 (0–6)	6 (2–8)	2 (0–4)	0.001*
FFP (ml)	800 (150–1287)	1200 (400–2400)	700 (0–1200)	0.02*
Platelets (u)	0 (0–2)	1 (0–5)	0 (0–1)	0.01*
*Outcome*				
Duration of ECMO (d)	11 (6–19)	9 (5–19)	12 (6–20)	0.66
ICU stay (d)	17 (10–35)	13 (11–27)	17 (8–34)	0.50
Hospital stay (d)	19 (11–42)	17 (11–42)	20 (10–39)	0.54
Survival (*n*, %)	38 (36%)	7 (30%)	31 (38%)	0.30

Bold indicates when P value is less than 0.05

*IQR* interquartile range

*P < 0.05

A total of 105 patients were included, with an average age of 49 (± 16) years; 76 patients (72%) were male. The most common cause of severe pneumonia was viral pneumonia (51%), followed by bacterial pneumonia (29%). Before ECMO, the APACHE II score was 18 (± 6) points, the SOFA score was 9 (± 4) points, the PRESERVE score was 5 (± 3) points, the RESP score was 2 (–1–4) points and the Murray score was 3 (± 1) points.

Compared with the non–BSI group, the BSI group a higher proportion of males (91% vs. 67%, *P* = 0.02), a higher proportion of patients with hypertension (52% vs. 28%, *P* = 0.03), and a higher Murray score [4 (± 1) vs. 3 (± 1), *P* = 0.02]. In addition, higher rates of blood transfusion (74% vs. 49%, *P* = 0.03), packed RBCs (pRBCs) [6 (2–8) vs. 2 (0–4) u, *P* = 0.001], fresh frozen plasma (FFP) [1200 (400–2400) vs. 700 (0–1200) ml, *P* = 0.02] and platelet (Plt) [1 (0–5) vs. 0 (0–1) u, *P* = 0.01] transfusion were identified in the BSI group. The number (and proportion) of patients with AKI receiving CRRT in the BSI group was significantly higher than that in the non–BSI group (70% vs. 43%, *P* = 0.02). There was no significant difference in age, BMI, smoking history, past medical history, ECMO duration, ICU stay, hospital stay or mortality rate between the two groups.

### Independent risk factors for BSI in ARDS–VV–ECMO patients (***Table ******2***)

**Table 2:** Independent risk factors for BSI in ARDS–VV–ECMO patients

Variables	OR	95% CI	*P* value
Male sex	4.68	0.85–25.71	0.08
History of hypertension	2.06	0.66–6.36	0.21
Murray score	6.29	1.71–23.10	0.01*
Transfusion	1.27	0.07–0.89	0.03*
CRRT	0.87	0.27–2.86	0.36
ECMO duration	1.01	0.98–1.04	0.39

Bold indicates when P value is less than 0.05

*OR* odds ratio;* CI* confidence interval;

*P < 0.05

Variables with a *P* value < 0.05 and relevant variables reported in previous studies, including male sex, history of hypertension, Murray score, transfusion, CRRT, and ECMO duration, were included in the multiple logistic regression analysis. The Murray score (odds ratio = 6.29, *P* = 0.01) and blood transfusion (odds ratio = 1.27, *P* = 0.03) were independent risk factors for BSI.

## Discussion

Many studies evaluating BSI during ECMO have been conducted in patients receiving VA–ECMO [9, 10, 12]. Among the few studies involving ARDS patients supported by VV–ECMO [1, 16], the etiology of ARDS variable and included interstitial lung diseases, radioactive pneumonia, diffuse alveolar hemorrhage, trauma and other diseases in addition to severe pneumonia. This study retrospectively analyzed the incidence and risk factors for BSI in patients with severe ARDS associated with pulmonary infection receiving VV–ECMO support. The etiology of ARDS was relatively singular, excluding other confounding factors.

The incidence of BSI was 22% in our study, which was similar to that in other studies [1, 16]. Our study showed that the most common pathogenic microorganism of BSI in severe ARDS patients with VV–ECMO was G^–^ bacilli, mainly *Acinetobacter baumannii*, *Burkholderia cepacia* and *Klebsiella pneumoniae*, consistent with the findings of some single–center studies [4, 17]. However, other researchers revealed that *Candida* (13%, the average incidence was 2.33/1000 ECMO days) and *Staphylococcus aureus* (7–10%, the average incidence was 1.20–2.37/1000 ECMO days) were the most common [18] isolates from BCs in patients receiving ECMO. We also observed that the incidence of BSI caused by MDR bacteria, especially multidrug–resistant G^–^ bacteria (MDR–GNB), was high during VV–ECMO support (34%). The high rate of antibiotic resistance is worrisome and can be attributed to frequent exposure to broad–spectrum antibiotics, acquired or primary immune impairment, prolonged hospitalization and mechanical ventilation [19, 20].

Although the association between blood transfusion and nosocomial infection in critically ill patients has been confirmed [21, 22], whether there is a potential association between blood transfusion and BSI in ECMO patients remains uncertain. Our study showed that blood transfusion was an independent risk factor for BSI, which was consistent with previous studies. Soo [23] et al. noted that the total units of transfused blood (aOR, 1.01; 95% CI, 1.00–1.02) was independently associated with BSI in patients receiving VV–ECMO for respiratory failure. Biologically, transfusion of pRBCs may increase the risk of BSI by interfering with the cytokine profile of the host. Several studies have shown that pRBCs contain multiple proinflammatory cytokines that, when infused into a susceptible subject, could potentially tip the balance and lead to the dysregulation of multiple cascades [24]. Thus, transfusion directly promotes inflammation, as demonstrated in studies that observed elevated levels of interleukin–6 in a recipient following pRBC administration [25]. On the other hand, residual donor white blood cells (WBCs) could promote T–cell activation [26], which in turn could result in subtle changes in the host's immune status, predisposing him or her to infection. Both cellular and humoral immunity are adversely affected by blood transfusion [27]. Following pRBC transfusion, decreased production of interleukin–2 and increased production of prostaglandin–E2 have been documented. A decrease in CD4 helper cells, interleukin–2 receptor–positive helper cells, and natural killer cells occurs, as well as an increase in B cells and CD8 suppressor cells. Some immune functions return to normal within hours following pRBC transfusion, but evidence suggests that long–term or permanent alterations in immune function may occur [27].

Most patients who receive ECMO support are in critical condition and are often complicated with AKI, with an incidence of 70.3%–84.4%. Approximately 60% of ECMO patients require CRRT [28]. In this study, 50% of ECMO patients received CRRT, while as many as 70% of patients with BSI required CRRT. The rate of CRRT in VV–ECMO patients with BSI was 48–83% in other studies [1, 10, 16, 17]. An injured kidney may compromise the immune response via systemic release of leukocytes from the kidneys and renal tubular cells. Such changes in the host immune response may be associated with nosocomial infection [29, 30]. CRRT in AKI may be an independent risk factor for BSI, which may be related to potential multisystem disorders, systemic inflammation, hormone imbalance [31, 32], prolonged duration of ECMO or CRRT–related operation in VV–ECMO patients [10].

Immunosuppression is a risk factor for infection. Nosocomial infection has been shown to be common in immunocompromised patients receiving ECMO support [19, 33, 34]. However, in some previous studies, there was no significant difference in immunosuppressive status between the BSI group and the non–BSI group [1, 16, 17], which was consistent with the results of our study. Our study found that immunosuppressive status did not increase the risk of BSI.

A previous study demonstrated that ECMO duration of more than 250 h significantly increased the incidence of BSI [5, 33]. However, some researchers believe that a prolonged duration of ECMO might be an adverse effect of BSI rather than a risk factor for BSI [16]. In our study, there was no association between ECMO duration and BSI.

Kutleša M [1] et al. reported that the incidence of massive hemorrhage was 34% in patients receiving VV–ECMO for ARDS, and massive hemorrhage was independently associated with BSI. The most common sites of massive hemorrhage are the gastrointestinal tract (GIT), urinary tract (UT), abdominal and thoracic cavities. Bleeding from the GIT, UT and nasal cavity can enhance the translocation of colonizing bacteria from those sites to the bloodstream. Furthermore, any significant bleeding requires urgent treatment with multiple invasive procedures, which might disrupt the mucosal barrier and increase the risk of infection. Finally, major bleeding episodes increase the duration of ECMO, which also increases the chances of acquiring BSI [4]. In our study, the incidence of massive hemorrhage events in the BSI group was 35%, and the most common types were GIT hemorrhage, cerebral hemorrhage and alveolar hemorrhage. We found no difference in bleeding events between the BSI group and the non–BSI group. The possible reasons were the strict aseptic techniques applied in all invasive operations and the administration of broad–spectrum antibiotics, which reduced the occurrence of BSI to some extent.

Our study has several limitations. It was conducted in a single clinical center and thus may lack general representativeness in terms of disease types and population. Our results should be confirmation in large–scale, prospective multicenter studies. In addition, the small number of BSI patients may lead to unreliable statistical conclusions. To accurately determine the source of BSI, strain homology analysis should be performed, but we did not conduct this analysis. However, this is one of the few studies conducted in BSI in patients with severe pneumonia–associated ARDS who received ECMO. This study systematically analyzed the incidence and risk factors in these patients.

## Conclusion

G^–^bacilli are the main pathogens causing BSIs in patients with severe ARDS supported by VV–ECMO, and most of them are MDR–GNB. The Murray score and blood transfusion may be independent risk factors for BSI.

## Data Availability

The datasets used and/or analyzed during the current study are available from the corresponding author upon reasonable request.
